# Tyrosine pathway regulation is host-mediated in the pea aphid symbiosis during late embryonic and early larval development

**DOI:** 10.1186/1471-2164-14-235

**Published:** 2013-04-10

**Authors:** Andréane Rabatel, Gérard Febvay, Karen Gaget, Gabrielle Duport, Patrice Baa-Puyoulet, Panagiotis Sapountzis, Nadia Bendridi, Marjolaine Rey, Yvan Rahbé, Hubert Charles, Federica Calevro, Stefano Colella

**Affiliations:** 1 Insa-Lyon, UMR203 BF2I, Biologie Fonctionnelle Insectes et Interactions, Villeurbanne, F-69621, France; 2 Inra, UMR203 BF2I, Biologie Fonctionnelle Insectes et Interactions, Villeurbanne, F-69621, France; 3 Université de Lyon, Lyon, F-69000, France; 4 Inria Rhône-Alpes, Bamboo, Monbonnot Saint-Martin, F-38330, France

**Keywords:** Symbiosis, Pea aphid, Metabolism, Development, Viviparous parthenogenesis, Amino acids, Tyrosine pathway, Cuticle formation, Microarrays transcriptome analysis

## Abstract

**Background:**

Nutritional symbioses play a central role in insects’ adaptation to specialized diets and in their evolutionary success. The obligatory symbiosis between the pea aphid, *Acyrthosiphon pisum,* and the bacterium, *Buchnera aphidicola,* is no exception as it enables this important agricultural pest insect to develop on a diet exclusively based on plant phloem sap. The symbiotic bacteria provide the host with essential amino acids lacking in its diet but necessary for the rapid embryonic growth seen in the parthenogenetic viviparous reproduction of aphids. The aphid furnishes, in exchange, non-essential amino acids and other important metabolites. Understanding the regulations acting on this integrated metabolic system during the development of this insect is essential in elucidating aphid biology.

**Results:**

We used a microarray-based approach to analyse gene expression in the late embryonic and the early larval stages of the pea aphid, characterizing, for the first time, the transcriptional profiles in these developmental phases. Our analyses allowed us to identify key genes in the phenylalanine, tyrosine and dopamine pathways and we identified *ACYPI004243*, one of the four genes encoding for the aspartate transaminase (E.C. 2.6.1.1), as specifically regulated during development. Indeed, the tyrosine biosynthetic pathway is crucial for the symbiotic metabolism as it is shared between the two partners, all the precursors being produced by *B. aphidicola*. Our microarray data are supported by HPLC amino acid analyses demonstrating an accumulation of tyrosine at the same developmental stages, with an up-regulation of the tyrosine biosynthetic genes. Tyrosine is also essential for the synthesis of cuticular proteins and it is an important precursor for cuticle maturation: together with the up-regulation of tyrosine biosynthesis, we observed an up-regulation of cuticular genes expression. We were also able to identify some amino acid transporter genes which are essential for the switch over to the late embryonic stages in pea aphid development.

**Conclusions:**

Our data show that, in the development of *A. pisum,* a specific host gene set regulates the biosynthetic pathways of amino acids, demonstrating how the regulation of gene expression enables an insect to control the production of metabolites crucial for its own development and symbiotic metabolism.

## Background

Symbiosis plays a key role in the life of many insects that live on nutritionally unbalanced diets, such as plant sap, blood or grain [1,2]. The adaptation of these insects to such food sources is possible only in association with certain microorganisms that are specialized in nutritional complementation. Endosymbiosis is, thus, a central process in these animals and more than 10% of insect species depend on intracellular bacteria for their development and survival [3]. This is true for aphids that feed on phloem sap [4], a very unbalanced diet that is characterized by a high concentration of sucrose and by very low levels of several essential amino acids crucial to the development of these metazoans [5]. The obligate intracellular symbiotic bacteria in aphids belong to the *Buchnera* genus and they provide these important pest insects with the essential amino acids lacking in their diet [6-10]*.* Symbiotic bacteria are contained in specialized host cells, called bacteriocytes, that are localised in the abdominal haemocoel, close to the ovaries in sexual and asexual females [1]. In fact, aphids have a life cycle that alternates sexual and asexual reproduction [11] and the success of aphids as crop pests is enhanced by their phenomenal reproductive rates, through viviparous parthenogenesis, during spring and summer. Parthenogenetic viviparous females have two ovaries, each containing several ovarioles. In the pea aphid, *Acyrthosiphon pisum,* embryos at different stages of development can be observed, at any given time, within six or seven ovarioles [12,13] and an adult asexual female reared in the laboratory produces an average of 130 embryos during her lifespan. The vertical transmission process of the symbionts is vital for the reproductive success of aphids and it takes place at the end of blastoderm formation in the *A. pisum* embryonic development [13,14]. At this stage, approximately 1000 *Buchnera aphidicola* bacteria are transmitted from maternal bacteriocytes to a single viviparous embryo, and they increase in number by 120 fold during the remaining embryonic development [1,13,15-17].

The importance of *B. aphidicola* nutritional complementation was initially indicated by the observation that aposymbiotic aphids (in which the symbiotic bacteria have been depleted using an antibiotic treatment) show significantly reduced growth and reproductive rates [18-22]. *A. pisum* has been extensively used in laboratory studies and its genome has been recently sequenced and annotated [23]. Several *B. aphidicola* genomes, from different aphid species [10,24-27] and from strains of pea aphid [28], have also been sequenced. In fact, a comparison between the pea aphid genome and that of its symbiont [10,28] confirmed the previously hypothesized integrated metabolism for these two organisms, in particular for the amino acid pathways [23,29,30]. The symbiotic bacterial genome includes genes for almost all enzymes involved in the essential amino acid pathways, while the few missing genes in the leucine, isoleucine, valine, methionine and phenylalanine pathways are present in the host genome [10,29]. A recent RNAseq transcriptome study of maternal bacteriocytes, compared with other tissues, supports the integrated nature of the host-symbiont metabolic network: this is demonstrated by specific gene expression regulations of amino acid metabolism genes in the symbiotic compartment, compared with other aphid body compartments [31].

The availability of the genome sequences for both partners of this symbiosis opens up several new research perspectives for this genomic model of symbiosis [32,33]. Functional genomics will help to characterize the role of different genes, and their regulation, in different physiological processes that contribute to the reproductive and ecological success of aphids. As seen in several other symbiotic bacteria, the *B. aphidicola* genome is reduced in size when compared to that of free-living bacteria [34] and it shows a clear reduction in the classic bacterial gene expression regulatory networks (reviewed in [35]). Several studies have indicated the lack of a strong and specific transcriptional response of this bacterium following a stress applied to the aphid host [36-38]. Nevertheless, a structured link between the organization of genes on the chromosome and gene transcription levels is conserved in *B. aphidicola,* as compared to the phylogenetically related free-living bacteria [39], and, more recently, a specific transcriptional response of the pLeu plasmid to a leucine stress applied to the pea aphid host has been demonstrated [40]. The integrated metabolism of the two partners indicates that the *B. aphidicola* regulatory capability is connected to the host transcriptional responses to stress events or to different physiological conditions. Certain studies have analysed the pea aphid transcriptome in different tissues and physiological conditions [38,41-47], but no global gene expression analysis of the aphids’ parthenogenetic embryonic development has yet been performed.

Although the metabolic complementation between *B. aphidicola* and aphids is important throughout their life cycle, the symbiosis is thought to play a key role during the parthenogenetic embryo development (reviewed in [48]). For example, embryonic growth in aphids is highly dependent on bacterial-derived phenylalanine and tryptophan and, to a lesser extent, on methionine and lysine [49]. After microinjections of radioactively labelled amino acids into the haemocoel of adult aphids, the selective uptake of phenylalanine and lysine by embryos confirms their specific metabolic needs [50]. It is difficult to determine whether this requirement for a high level of amino acids in pea aphid embryos is supported by the maternal tissues (maternal bacteriocytes and/or other tissues) or by the embryonic complement of the symbiotic bacteria localized in the embryonic bacteriocytes. It has been established that late embryos can rely on their own complement of symbiotic bacteria for a supply of essential amino acids, particularly for the aromatic amino acids [51]. More recently, a study on the embryos of the black bean aphid *Aphis fabae*, maintained *in vitro* with artificial diets lacking amino acids, confirmed the importance of certain amino acid supplies, specifically phenylalanine and valine, acquired exogenously from *B. aphidicola* in the maternal symbiosis, and tryptophan, acquired endogenously from symbiotic bacteria within the embryo [52].

In all the early *ex vivo* studies of embryonic nutritional needs, the ovaries were always analysed as a whole and, consequently, no data on the distinct embryonic developmental stages were available. More recently, a study on the separate stages was performed to assess *B. aphidicola* transcriptional changes during development [53]. This work revealed that the number of *B. aphidicola* genes differentially expressed between embryos and maternal tissues varies significantly among the early, intermediate and late embryos, indicating that the symbiotic interactions between the developing host and its bacterial partner are dynamic, changing in response to the developmental age of the host. The availability of the *A. pisum* genome prompted us to complement this work with an analysis of pea aphid transcription profiles during parthenogenetic development. Using a NimbleGen custom microarray, we analysed the host transcriptome comparing different developmental groups, thus generating a complete analysis of gene expression during viviparous parthenogenesis of a symbiotic insect. These transcriptomic data were coupled with an analysis of amino acid metabolism, which enabled us to identify some key aspects of the contribution of the symbiotic partners to the metabolic needs of developing parthenogenetic embryos.

## Results

### Global analysis of gene expression during embryonic development

**Figure 1:**
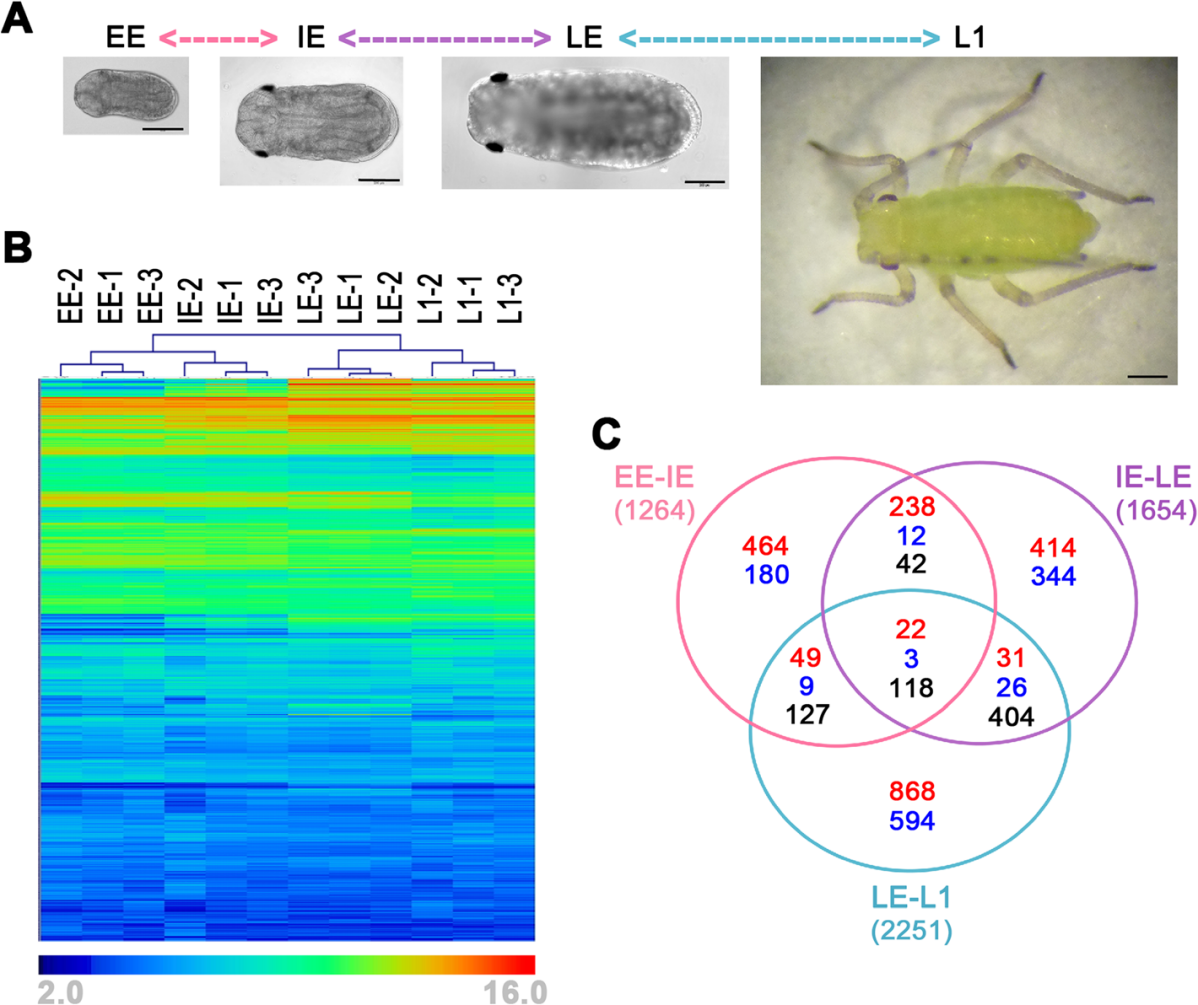
**Global expression analysis in pea aphid development. A**) Micro-photographs of the four stages analysed in this work where the scale bar represents 200 μm in all photographs to allow for size comparison. The microphotographs show just one embryo stage among those belonging to the corresponding groups (see Table 1 for detail). The performed comparisons, early embryos (EE) *versus* intermediate embryos (IE), intermediate *versus* late embryos (LE) and late embryos *versus* first larval stage (L1) are labelled, respectively, in rose, violet and light blue. **B**) Unsupervised hierarchical clustering (generated by an average linkage method with euclidean distance and no leaf order optimization) of 50% of transcripts (12,005) showing the higher standard deviation among all the samples. The colour chart indicates expression intensities using a base 2 logarithmic scale: blue and red represent, respectively, lower (2.0) and upper (16.0) expression intensities (see bottom panel legend). **C**) The Venn diagram of the 3945 genes showing significantly differential expression in the three comparisons (comparison colour code as in Figure 1A). Numbers in red identify the genes increasing in expression during development; in dark blue, the genes decreasing in expression during development; and, in black, the genes found to be significant in two or more groups where the expression changes are not varying in the same direction in the different comparisons.

Using the newly developed “INRA-BF2I_A.pisum_Nimblegen-ACYPI_4x72k_v1” microarray (ArrayExpress design ID: A-MEXP-1999), built on the pea aphid genome v1.0 assembly [23], we obtained gene expression profiles of aphid embryos belonging to three distinct groups, namely early (EE), intermediate (IE) and late (LE) embryos, collected according to their developmental stage (see Table 1 and Figure 1A), together with aphids at their first larval stage (L1). Three biological replicates were used for each experimental group (see Methods section for further details).

**Table 1:** Description of embryonic and larval stages used for the transcriptomic and amino acid content analyses

	**Group**	**Group abbreviation**	**Developmental stages**	**Size (length or weight)**	**External morphological features**
**Embryos**	Early embryos	EE	0-15	≤ 400 μm	*No visible eyes *Very slight body pigmentation
	Intermediate embryos	IE	16-18	400-800 μm	*Developing eye spots in many individuals *Pigmented bodies
	Late embryos	LE	19-20	> 800 μm	*Developed eye spots in all individuals *Highly pigmented bodies
**Larvae**	First instar larvae	L1	1^st^ larval	≤ 0.2 mg	0-24 hours old
		L1 early			≤ 6 hours old
		L1 late			≥ 15 hours old

Assignment of embryos to the three groups (EE, IE or LE) was based on size range and on morphological criteria detailed in this table (see Figure 1A for exemplary microphotographs). The developmental stages were defined according to Miura *et al.*[13]. Larval stages were determined according to the development time after laying.

To check the overall quality of the data, after normalization, we performed an unsupervised cluster analysis using the 50% of genes showing the highest SD in all the samples (*i.e.* 12,005 genes) and, with this approach, we were able to classify the data in the original 4 groups analyzed (Figure 1B), with all the biological replicates clustering together. As this analysis confirmed the reliability of our data following the dissection and isolation of the embryos, we then used all the data available to perform the following pair-wise comparisons: (a) EE *vs.* IE, (b) IE *vs.* LE and (c) LE *vs.* L1, using a one-way between groups ANOVA. Among the 24,011 transcripts analyzed, 3,945 (16.4%) were considered as being significantly differentially expressed during the development of the pea aphid using the following criteria: an adjusted p-value lower than 0.05 and a two-fold change in the considered contrast (see Methods for details of the analysis). Using these criteria, we identified, respectively, 1,264, 1,654 and 2,251 differentially expressed genes for the comparisons EE-IE, IE-LE and LE-L1 (Figure 1C and Additional file 1: Table S1). We observed an increase in the total number of genes differentially expressed during development, whereas the proportion of up-regulated genes decreased by 78.6%, 66.0% and 51.3%, respectively, in the three sequential comparisons EE-IE, IE-LE and LE-L1. This observation shows an activation of the expression of many genes in the earlier stages (comparison EE *vs*. IE), followed by a down-regulation of the genes important for development and not needed for the first instar larval stage aphids (L1). An analysis of the intersection between stages allowed us to characterize the developmental switches and to identify significant expression changes. For example, the highest number of common genes is found at the intersection between the IE-LE and LE-L1 comparisons, with 604 genes (Additional file 1: Table S1D). It is worth noting that, out of these 604 genes common to the IE-LE and LE-L1 comparisons, 516 showed expression changes in opposite directions in the two comparisons and, among those, 366 were up-regulated between IE and LE and down-regulated between LE and L1. These observations support the fact that numerous genes that are important in development are activated in the early stages and they are gradually repressed in the more advanced stages, up to the L1 stage. On the other hand, between the EE-IE and IE-LE comparisons, we observed a continuity of expression in the majority of common genes (340 out of 435 changed in the same direction, with 322 showing an up-regulation during development). Overall, our results show the expected higher number of genes changing their expression at the transition between the embryonic and larval stages.

### Microarray data validation by quantitative RT-PCR

To validate the data obtained from the microarray analysis in our first experiment, we repeated the experiment for the same four stages and quantified the expression of eight *A. pisum* genes, belonging to four functional classes (3 developmental genes, 3 amino acid pathways genes, 1 cuticular gene and 1 transporter gene), using quantitative reverse transcription-PCR (qRT-PCR). The whole experiment was performed again using three new biological replicates for each stage group and for these samples total RNA was not amplified (see Methods for details). We compared the data obtained from qRT-PCR with that obtained in the independent microarray experiments and found very good concordance, with a Pearson’s correlation coefficient of 0.87 (p < 0.0001) between these two datasets (Additional file 2: Table S2) [54].

### Developmental genes expression analysis

**Figure 2:**
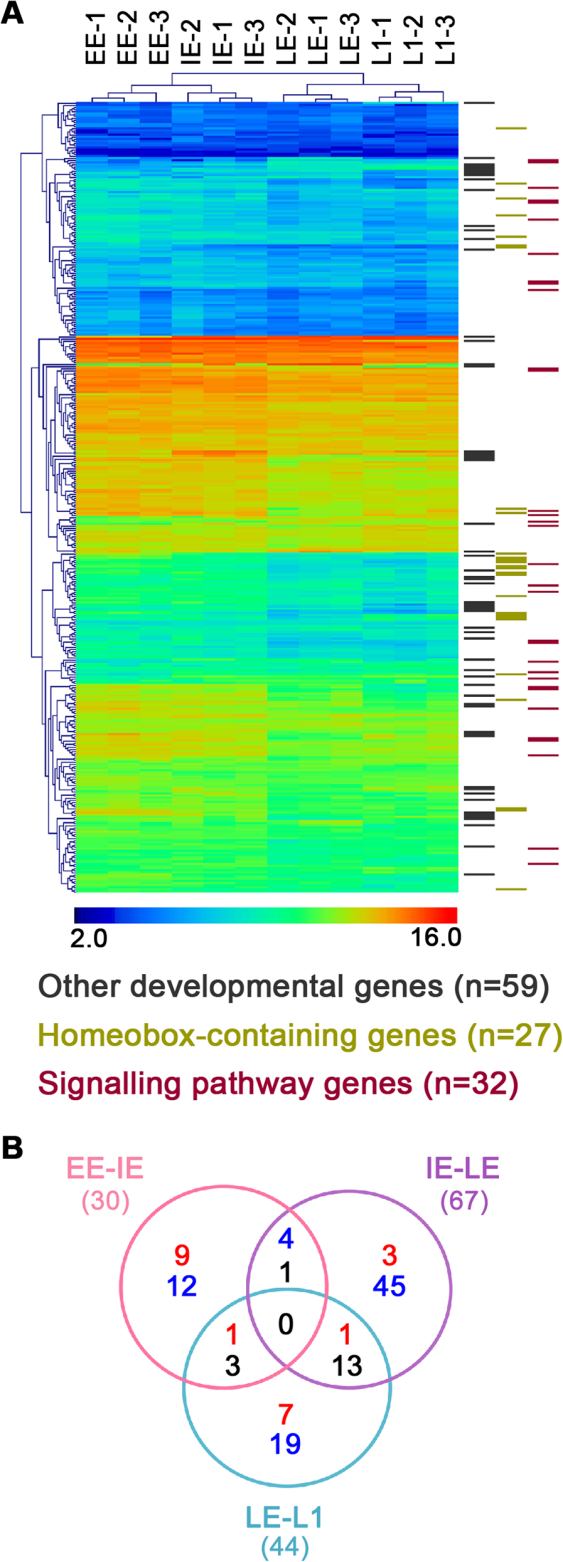
**Developmental gene expression analysis in pea aphid. A**) Hierarchical clustering (generated by an average linkage method with euclidean distance and no leaf order optimization) on the 368 developmental genes present on the microarrays (out of the 387 annotated by Shigenobu *et al.*[55]). The colour chart indicates expression intensities using a base 2 logarithmic scale: blue and red represent, respectively, lower (2.0) and upper (16.0) expression intensities (see bottom panel legend). On the right of the cluster, genes showing significant differential expression in at least one of the comparisons are indicated: in green, 27 homeobox-containing genes; in red, 32 signalling pathways genes; and, in black, 59 other developmental genes. **B**) The Venn diagram, showing the 118 developmental genes with significantly differential expression in the three comparisons (comparison colour code as in Figure 1A) analysed in this study. Numbers in red identify the genes increasing in expression during development; in dark blue, the genes decreasing in expression during development; and, in black, the genes found to be significant in two or more groups where the expression changes are not varying in the same direction in the different comparisons.

Among the 387 pea aphid developmental genes annotated by Shigenobu *et al.*[55], using homology with *Drosophila melanogaster,* 368 were present in our microarrays and were analysed. In our dataset, 118 genes (32%) showed significant differential expression in at least one of the three comparisons we performed (Figure 2A). In the comparisons EE-IE, IE-LE and LE-L1 we found, respectively, 30, 67 and 44 significant variant genes (Figure 2B and Additional file 3: Table S3). Twenty-one genes increased their expression during development, while 80 genes showed decreasing levels of expression. Among these 80 genes, 45 were found only in the comparison IE-LE, *i.e.* the last of the embryonic developmental stages analyzed in this study. We performed a detailed analysis of two gene classes, as defined in the Shigenobu *et al* annotation paper [55]: the homeobox-containing genes and the signalling-pathway genes, which are involved in the establishment of anatomical patterns and in the regulation of developmental processes in all the metazoans. Among the 95 homeobox-containing genes annotated in the genome of *A. pisum,* 27 were identified as differentially expressed in at least one of the three comparisons; the same was true for 32 signalling pathway genes, among the 101 annotated, showing significant differential gene expression at different stages of development (Figure 2A and Additional file 3: Table S3D). We also performed a detailed manual analysis for 80 genes belonging to the six main classes of developmental genes classified as being involved in body axis formation, in embryo segmentation, in germline specification, in neurogenesis and in eye development, together with the Hox genes. Of these 80 genes, only 18 showed differential expression in at least one comparison. None of these genes were up-regulated in the EE-IE and IE-LE comparisons and the majority were down-regulated during development, when comparing IE with LE. A few genes showed up-regulation in the LE to L1 transition, including homologs of the *D. melanogaster Tslr, Knrl-1, ci, Hh, Ubx* and *so* genes (Additional file 3: Table S3).

### Gene ontology analysis of significant genes

We performed an unsupervised analysis of the genes showing significant differential expression in the three comparisons (EE-IE, IE-LE and LE-L1), using an enrichment analysis of the functional classes of genes based on the Gene Ontology (GO) annotation. This analysis revealed 246, 274 and 94 enriched functional classes for the comparisons EE-IE, IE-LE and LE-L1, respectively, thus showing a higher number of gene class changes in the two embryonic development comparisons (Additional file 4: Table S4). We observed enrichment in transporter activity genes both in the EE-IE and IE-LE comparisons, with the presence of amino acid transporters being amongst the most significant. In the IE-LE comparison, the morphogenesis and appendage development genes showed significant changes in expression levels. For the IE-LE and LE-L1 comparisons, the analysis revealed three GO classes involved in the cuticle formation process.

### Metabolism gene expression: the amino acid pathways

To explore the general metabolic changes that take place during the parthenogenetic development of the pea aphid, we analyzed the relevant genes (Additional file 1: Table S1) using the annotations available in the AcypiCyc database, which contains the global reconstruction of the metabolic network of the pea aphid [56]. At least one enzyme-coding gene showed significant differential expression for 43, 74 and 90 pathways in the EE-IE, IE-LE and LE-L1 comparisons, respectively (Additional file 5: Table S5). We classified all these pathways, in AcypiCyc, into five broad groups of compounds: lipids, amino acids, sugars, nucleotides, and others. An analysis of the distribution of genes within these classes, compared with the total number of genes in the pea aphid genome, did not reveal any significant changes that would indicate a class of particular interest. For example, 16.8% of the total number of differentially expressed enzyme-coding genes in our dataset forms part of the amino acid metabolism group and, in the pea aphid genome, the percentage of genes coding for this class is 16.4%. So, the number of detected genes is not significantly different from the number that would be expected by chance. The same is true for the other classes, but we decided to carry out further gene-by-gene analysis for those classes supposed, *a priori,* to play an important role in symbiosis or in developmental processes.

**Figure 3:**
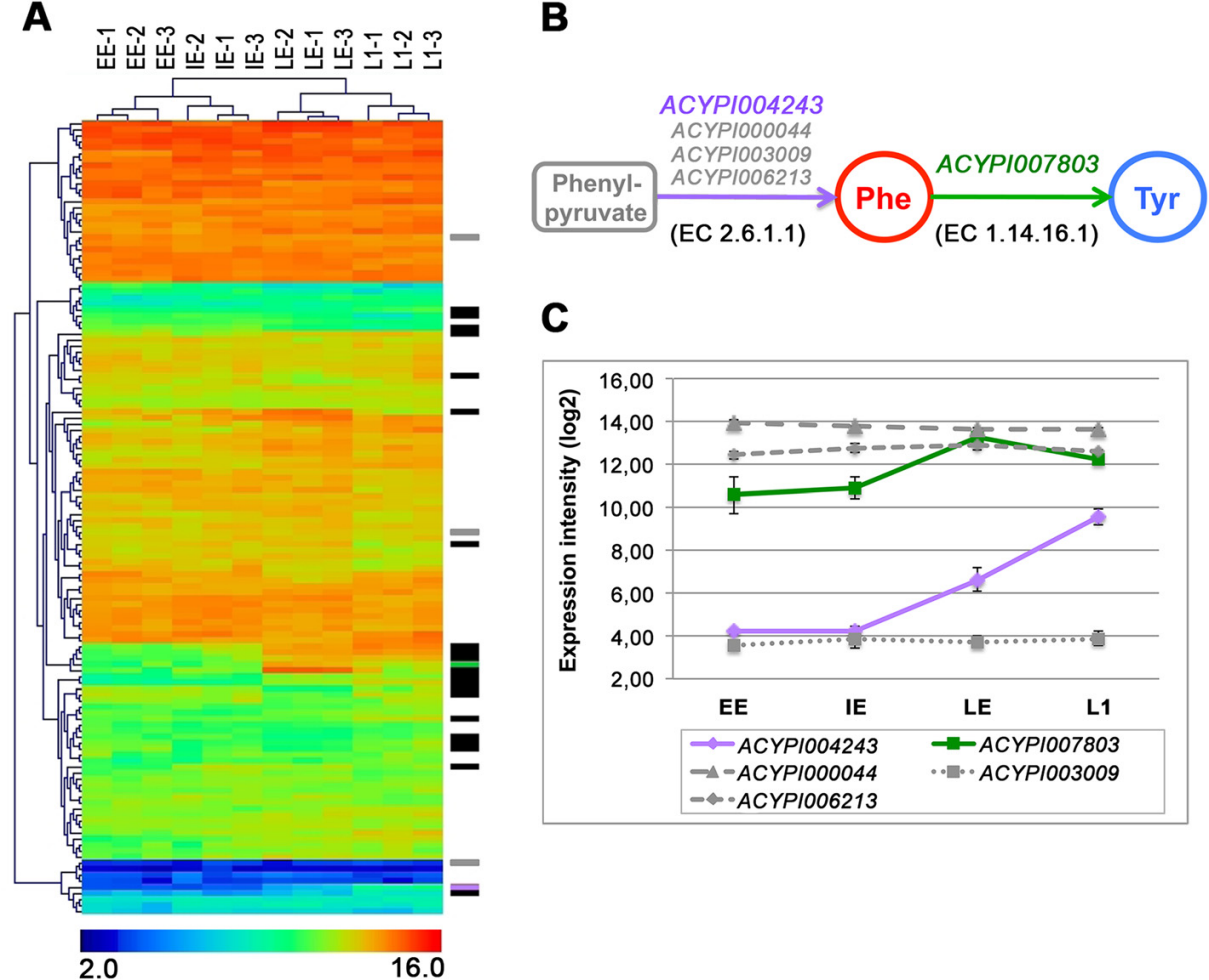
**Amino acid metabolism gene expression profiling during pea aphid development. A**) Hierarchical clustering (generated by an average linkage method with euclidean distance and no leaf order optimization) of the 135 amino acid metabolism genes annotated by Wilson *et al.*[29]. The colour chart indicates expression intensities using a base 2 logarithmic scale: blue and red represent, respectively, the lower (2.0) and upper (16.0) expression intensities (see bottom panel legend). On the right of the cluster we reported: in black, the genes varying significantly in at least one of the comparisons, with the exception of the ACYPI007803 gene, shown in green, and ACYPI004243, shown in purple. The three other genes coding the 2.6.1.1 enzyme activity are highlighted in grey but do not vary significantly during development. **B**) Final steps of the pathway for phenylalanine and tyrosine biosynthesis encoded in the pea aphid genome. **C**) Expression profiles of the genes involved in the pathway for phenylalanine and tyrosine biosynthesis in the pea aphid. Expression intensity is given as log2. Expression intensities of each stage are means of the three biological replicates.

In particular, we analyzed in more detail the gene expression profiles of the 135 genes involved in the amino acid biosynthesis or degradation pathways [29], which were present in our overall analysis and which represent one of the key functions in the physiology of pea aphid/ *B. aphidicola* symbiosis*.* This analysis revealed that the majority of the genes involved in these pathways are strongly expressed (Figure 3A): 78.5% of these genes are among the 25% of genes showing the strongest expression. Of the 135 annotated genes, 23 showed significantly differential expression in our comparisons (with 17 genes increasing and four decreasing in expression and two variations of expression in the opposite direction during development). Among the genes involved in amino acid biosynthesis, the significant ones can be classified into four groups: (1) the aspartate biosynthetic family (Asp, Asn, Met, Cys, Thr, Ile, Lys); (2) the phosphoenolpyruvate biosynthetic family (Phe, Tyr, Trp); (3) the glutamate biosynthetic family (Glu, Gln, Pro, Arg); and (4) the 3-phosphoglycerate biosynthetic family (Ser, Cys, Gly) (Additional file 6: Table S6).

The tyrosine (Tyr) pathway appeared to be particularly interesting with two genes, encoding for the proteins with the enzymatic activities EC 1.14.16.1 and EC 2.6.1.1, showing significantly increased levels of expression during development (Figures 3B and 3C). In particular the expression of the *ACYPI007803* gene, which encodes for the enzyme phenylalanine 4-monooxygenase responsible for the synthesis of Tyr in the pea aphid (EC 1.14.16.1), significantly increased in the IE-LE comparison (log2 difference of 3.36 for the IE-LE comparison). Another three genes were annotated as potentially coding for this enzyme: for two of them (*ACYPI000847* and *ACYPI008168*) other EC annotations were supported by the analysis in AcypiCyc (EC 4.2.1.96 and EC 1.14.16.2 respectively), while *ACYPI000175* had a weak annotation score for the enzymatic activity EC 1.14.16.1 but it did not show significant changes in expression.

Four genes in the pea aphid genome (*ACYPI000044, ACYPI006213, ACYPI004243*, and *ACYPI003009*) encode for the enzyme aspartate transaminase (EC 2.6.1.1), which catalyzes the synthesis of phenylalanine from phenylpyruvate (Figure 3B). Our gene expression analysis revealed 3 distinct transcription profiles for these four genes (Figure 3C): *ACYPI006213* and *ACYPI000044* showed a consistently high level of expression during development, *ACYPI003009* gene expression was constant and very low during development, while *ACYPI004243* showed a significant change in its expression level. The expression of this gene was relatively low in the early (EE) and intermediate (IE) embryo stages, compared to other genes, but it increased significantly between IE and L1 (2.37 and 2.91 log2 differences for the IE-LE and LE-L1 comparisons, respectively). The gene expression changes, detected using the microarrays for *ACYPI007803* and *ACYPI004243,* were also confirmed by specific qRT-PCR experiments (Additional file 2: Table S2).

### Amino acid transport activation

We analyzed, in detail, the amino acid transport function using the recent annotation by Price *et al*. [57]: our microarray contains probes for 28 of the 40 annotated amino acid transporters and four of the six annotated Na/K/Cl co-transporters of the pea aphid genome. All of the 32 genes represented on the array were detected as expressed in all the samples analyzed. Furthermore, as expected from the GO enrichment analysis, the majority (20 out of 32) of the genes coding for amino acid transporters are part of the significant group in at least one of the three comparisons (Additional file 7: Table S7). Among those genes, six out of 13 members of the eukaryotic specific amino acid/auxin permease (AAAP) family showed significant differential expression during development. With regard to the genes coding for the transporters of the amino acid/polyamine/organocation (APC) family, 11 out of 15 of them showed significant changes in gene expression levels during development. As for the Na/K/Cl co-transporters, needed to create the gradient used by the APC and AAAP transporters, three out of four represented on our microarray showed changes in gene expression. Our data constitute the first characterization of the transcription of amino acid transporter genes during pea aphid development and we found that not all of the genes belonging to the same specific gene family showed differential gene expression. For example, this is the case for the members of the pea aphid slimfast transporter gene family [57] that showed developmental stage-specific expression profiles (Additional file 7: Table S7).

### Accumulation of tyrosine in late embryo stages

**Figure 4:**
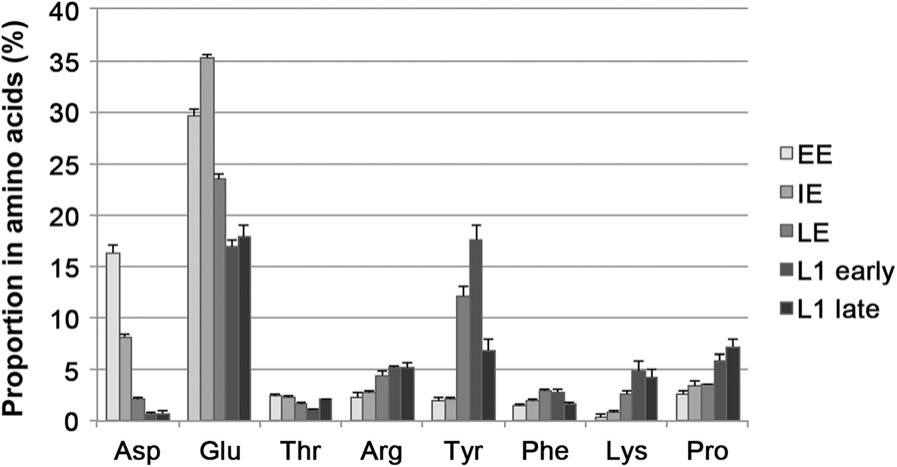
**Proportion of free amino acids during pea aphid development.** Free amino acid contents of early, intermediate and late embryos, and in the first larval stage taken at two distinct times of development (early L1 ≤ 6 hours and late L1 ≥ 13 hours). Values (n = 4) are expressed as a percentage of the total amount of amino acids in the different samples. Among the 18 amino acids analysed, only those varying the most (P < 0.0001) and phenylalanine (P = 0.0015), the precursor of tyrosine, are represented. In particular among the significant changes (see Table S8 for details) it is worth noting: a decrease of aspartic acid from 16.3% in the early embryos (EE) to 0.7% in L1 larvae; a glutamic acid decrease from IE (35.2%) to the L1 stages (around 17.4%); an accumulation of tyrosine during development from 1.9% in early embryos (EE) to 12.1% in the late embryo group (LE), accumulation that continues in early first larval stage (17.6%) to decrease rapidly to 6.8% for the late larvae L1.

We extended the analysis to metabolism by performing free amino acid analysis, using HPLC, on the same embryo groups used in the transcriptome experiments. To analyse the larval stages, on the basis of our transcriptome results, we decided to perform the analysis at two distinct time points of the first larval stage of development in order to monitor, in more detail, any changes in the amino acids content related to cuticle changes in larval growth. Therefore, our HPLC analysis on L1 larvae was performed on two groups: early L1 (age ≤ 6 h, including larvae at the very beginning phases of the cuticle maturation after birth) and late L1 (age ≥ 15 h, preparing the cuticle changes for the switch over to the L2 larval stage). The results of this analysis were expressed as a fraction of the total free amino acid content (Additional file 2: Table S8). For histidine and methionine, the concentrations in the EE and IE stages were below the method detection limits. For two amino acids (asparagine and leucine) no significant variation in proportion was observed between any of the stages. The aspartic and glutamic acids (both p < 0.0001) showed a tendency to decrease their relative concentration during development (Figure 4). Threonine decreased during embryonic development and increased in the larval stages. Four amino acids showed a significant increased relative concentration during development (p < 0.0001): arginine, tyrosine, lysine and proline (Figure 4). Among these amino acids, the greatest difference during development was observed for tyrosine in the late embryo group (LE), showing a six-fold increase in concentration compared with the early embryos (EE). The proportion of free tyrosine decreased rapidly form early to late larvae L1. However, phenylalanine, the precursor of tyrosine, did not show any significant change between any of the stages (Figure 4).

### Embryo development specific regulation of the gene *ACYPI004243*

The matching profiles of the enzyme-coding gene expression for tyrosine synthesis and the accumulation of this amino acid in the LE and L1 embryo groups prompted us to perform a more detailed analysis of the genes involved in this pathway (Figure 3B and 3C). To gain a better understanding of the differences between the four genes (*ACYPI000044, ACYPI003009, ACYPI004243*, and *ACYPI006213*), annotated in the AcypiCyc database as coding for the enzyme aspartate transaminase (EC 2.6.1.1), we performed a detailed enzyme gene annotation using the dedicated PRIAM tool [58]. This analysis revealed that, for all of the corresponding proteins, after the primary annotation as enzymatic activity aspartate transaminase (EC 2.6.1.1), two other complementary annotations follow: the enzymatic activity tyrosine transaminase (EC 2.6.1.5) and the enzymatic activity aromatic amino acid transaminase (EC 2.6.1.57) (Additional file 2: Table S9). This additional *in silico* analysis confirmed the hypothetical role of these four genes in coding the enzymes catalyzing the aspartate, tyrosine and aromatic amino acids transamination reactions. The two more specific annotations of PRIAM are not included in the AcypiCyc database as they did not pass the cut-off point applied in the generation of the database using CycADS [56]. Finally, we analyzed the gene structure and the genome organization of the four genes encoding for the enzyme aspartate transaminase (E.C. 2.6.1.1) in the pea aphid. Among these genes, *ACYPI000044* shows a two-exon structure, with the coding sequence restricted to exon 2, while all the other genes have an 8-exon structure (with the coding sequence spread out between exon 1 and exon 8). It is worth noting that the genes *ACYPI004243* and *ACYPI003009* mapped to the same contig in the pea aphid genome. An analysis of the protein sequences was performed and the alignment of the four proteins revealed the expected conservation, with the exception of the ACYPI000044-PA protein sequence that has a unique N-terminal portion which is not aligned to the other three proteins with the enzymatic activity aspartate transaminase (EC 2.6.1.1) (Additional file 8: Figure S1). A detailed phylogenetic analysis of the four aspartate transaminases, expanding the protein information available in PhylomeDB [59] and using the UniProt database [60], was performed (Additional file 9: Figure S2). The evolution of the aspartate transaminases, also called aspartate amino transferases (AAT) family, revealed five major duplications separating bacterial AAT (TyrB and AspC) from mitochondrial AAT (AATM or GOT2) and cytoplasmic eukaryote AAT families (AATC or GOT1). ACYPI000044-PA clearly belongs to mitochondrial AATM. ACYPI006213-PA, ACYPI004243-PA and ACYPI003009-PA diverged more recently and seem to be specific to aphids.

The peculiarity of the *ACYPI000044* gene structure, the differences observed in its protein sequence, and its phylogenetic position as regards the mitochondrial AAT prompted us to carry out an additional analysis of the ACYPI000044-PA unique N-terminal sequence. We can confirm that this corresponds to the mitochondrial targeting signal peptide. As a result, the ACYPI000044-PA protein has the highest score for potential export to mitochondria, when compared to the other three genes encoding for this enzymatic activity (Additional file 2: Table S10). The gene structure of 23 of the 26 AATM proteins, in the same clade as ACYPI000044-PA, was available (Additional file 9: Figure S2) and it is interesting to note that only two other genes had a single exon encoding the protein: these two genes are from the yeast *Schizosaccharomyces pombe* and the amoeba *Dictyostelium discoideum*.

### The cuticle formation pathway

Tyrosine is used to produce DOPA and dopamine, both of which are needed for melanization and sclerotization of the cuticle. Our observation of the increase in cuticle related gene expression between the LE and the L1 stages, coupled with the role of tyrosine as a precursor of cuticle formation processes, prompted us to analyse the DOPA and dopamine synthesis pathway in more detail. In effect, the gene coding for tyrosine 3-monooxygenase (*ACYPI008168*), the enzyme catalyzing the reaction converting tyrosine to DOPA (EC 1.14.16.2), showed significant up-regulation in the comparison IE *vs.* LE and a reduction in expression in the passage from LE to L1 (Additional file 10: Table S11A). Multiple pea aphid proteins have been annotated as having the enzymatic activity DOPA decarboxylase (EC 4.1.1.28) in AcypiCyc and, among them, the gene *ACYPI009626* received the highest annotation score [56]. It was also seen to be significantly up-regulated in the LE embryo group, compared to IE, and down-regulated in the transition from LE to L1 (Additional file 10: Table S11A). Another six proteins have been annotated as having the enzymatic activity aromatic-L-amino-acid decarboxylase (EC 4.1.1.28) but with a lower score [56]: five of them did not show any change in gene expression during development, while one showed a reduction in expression in the comparison LE vs. L1 (Additional file 10: Table S11A).

We also analyzed the 74 genes annotated as cuticular proteins by the GO annotation and, as expected from our Gene Ontology analysis (GO:0042302), the majority (64 out of 74) showed significant up-regulation during at least one stage in the late developmental phases (IE and LE samples), when the cuticle is being formed. Many of these genes (38 out of 64) showed a significantly lower level of expression in L1, when compared to LE, but others maintained a constant level of expression throughout the subsequent cuticular processes in larval development (Additional file 10: Table S11B).

**Figure 5:**
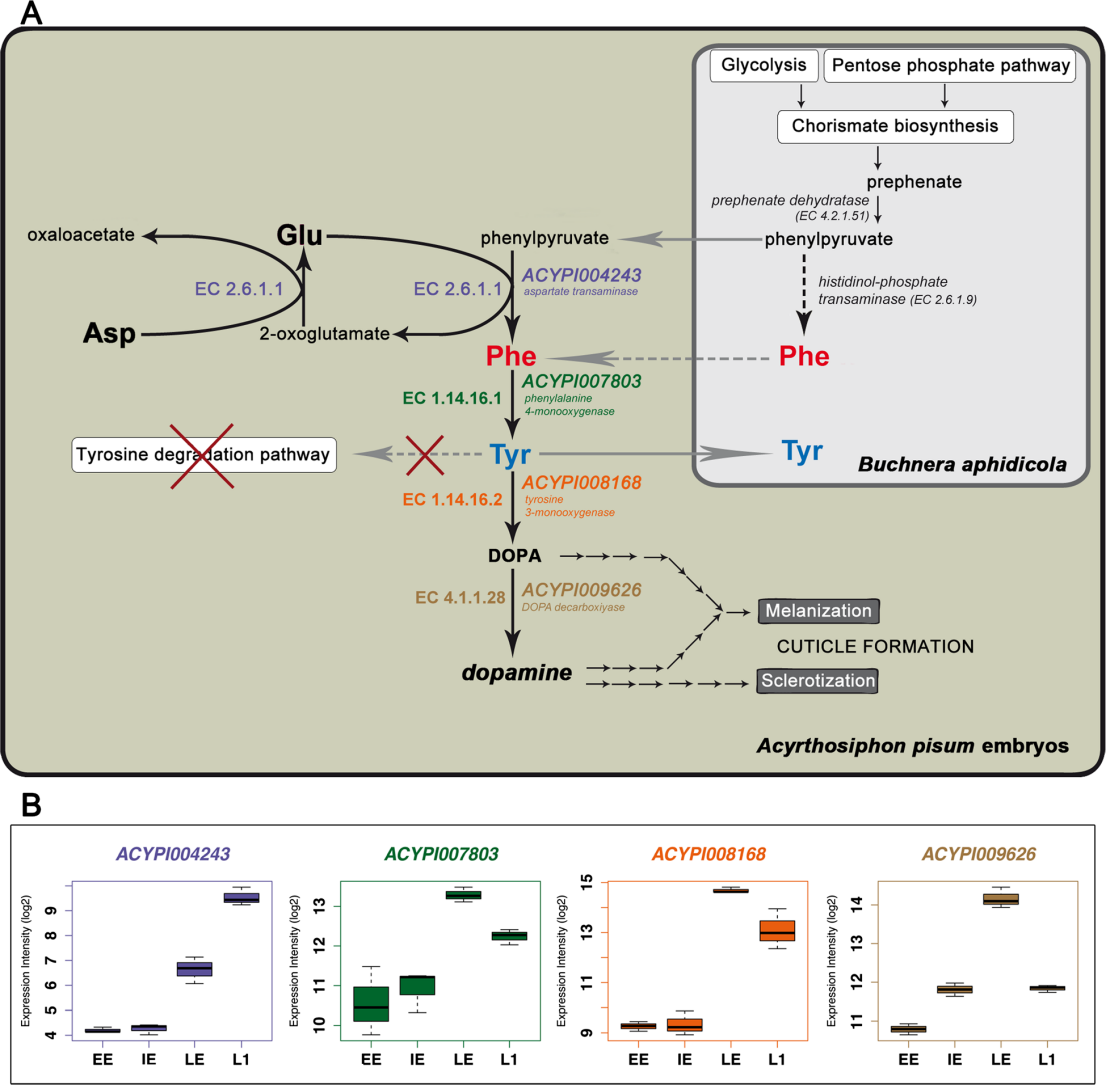
**Gene expression regulations acting on the integrated pathway for tyrosine and other precursors of the cuticular tanning biosynthesis in a pea aphid embryo associated with *B. aphidicola*. A**) Schematic representation of the pea aphid embryo (green rectangle) and its symbiotic bacteria (grey rectangle). A summarizing diagram of the Tyr/dopamine pathway during pea aphid development is represented. Black arrows represent reactions, grey arrows transport reactions; solid arrows are annotated events; dotted arrows are possible events; red crosses indicate events not present in the annotation of the genome (see text for details). Only the pea aphid genes whose expression levels change significantly during development are represented. The two *B. aphidicola* genes did not show significant expression changes during pea aphid development [53]. **B**) Expression profiles of the genes involved in the Tyr/dopamine pathway in the pea aphid. Expression intensity is given as log2. Expression intensities of each stage are means of the three biological replicates.

These data, taken together, demonstrate an up-regulation of the metabolic pathways leading to the synthesis of cuticular proteins and of the key precursors for the process of cuticle melanization and sclerotization, in the LE stage of pea aphid development (Figure 5 and Additional file 10: Table S11).

## Discussion

In this study, the transcriptome analysis of parthenogenetic development in embryonic pea aphids enabled us to characterize the gene expression profiles at key stages in the development of this insect. A comprehensive transcriptome dataset, such as this, is important for the ongoing genome annotation effort as gene expression profiles at specific steps of pea aphid development, or in specific tissues, can lead to an initial characterization of the role of different genes in aphid physiology.

After an unsupervised clustering analysis, that indirectly demonstrated the good quality of our dataset, we identified 3945 genes that showed significant changes in expression level in the developmental groups analysed here (Figure 1). We examined this high number of variant genes, combining unsupervised and hypothesis driven analyses.

We identified the expression profiles of key developmental genes among the ones that had been annotated, *in silico,* during pea aphid genome analysis [23,55] (Figure 2). Using a global analysis based on gene ontology (GO), we identified biological and cellular processes for which significant expression changes were apparent in the developmental stages under study. Between the IE and LE stages, several significant expression changes in the GO classes are linked, as expected, to the development of appendages and morphogenesis. In both the EE *vs.* IE and IE *vs.* LE comparisons a set of GO classes linked to amino acid transport were identified as changing significantly during development. To further investigate this observation, we analysed the set of pea aphid amino acid transporters recently characterized [57] and we demonstrated a global induction of the majority of these annotated transporter genes during development (Additional file 7: Table S7). These observations represent the first characterization of gene expression profiles of this family of genes involved in amino acid transport during the pea aphid’s parthenogenetic development. An intriguing amplification of ortholog genes of the *D. melanogaster slimfast* gene (member of the APC family) has been described in the pea aphid [57] and we have analyzed the gene expression profiles of nine out of the 10 gene copies of this insect genome. Seven of these *slimfast* orthologs showed increased expression during development in the stages where embryonic growth is greatest (Additional file 7: Table S7B): this observation is in agreement with the role of these genes as nutrient sensors during insect growth [61,62]. Among these *slimfast* genes showing increasing expression during parthenogenetic development, we find the three paralogs previously identified as having an expression bias in male morphs (*ACYPI003240, ACYPI005156* and *ACYPI002633*) [63]. This observation does not contradict the male-biased expression, but further characterization of these genes would be interesting to gain a better understanding of their role in the growth of the different morphs. We can speculate that the expansion of this gene family in the pea aphid genome, and the increased expression of several of these genes during embryonic development, might play a role in the high growth rate observed for parthenogenetic viviparous embryos in this insect. This hypothesis could be confirmed by transcriptional analysis indicating either lower gene expression levels or different regulations for the slimfast genes in embryos produced by sexual reproduction and having a low growth rate.

The recent pea aphid genome analysis showed how the enzyme repertoires of the two partners of this metabolically obligatory symbiosis are complementary [23]. A comprehensive analysis of the genes involved in metabolism, performed using the AcypiCyc database annotations [56], did not reveal any significant global change in gene expression in the specific pathways. Nevertheless, the known central role of amino acid metabolism pathways in this symbiotic relationship [29] and, in particular, during pea aphid development [48], together with the induction of expression of the amino acid transporter genes observed in our experiments, prompted us to analyse, in more detail, all the genes involved in the amino acid pathways [29]. Most genes coding for enzymes involved in amino acid metabolism showed strong expression levels (Figure 3), as expected given the central role of amino acids in embryonic development [49-52]. A significant induction of gene expression during pea aphid development, in the stages under study, affects several amino acid pathways (Additional file 6: Table S6). Among these, the pathway for the synthesis of phenylalanine and tyrosine showed significant gene expression changes in both of the enzymatic activities (EC 2.6.1.1 and EC 1.14.16.1) encoded in the pea aphid genome, for the steps complementary to those in *B. aphidicola* (Figure 5). To further investigate this pathway, which plays a key role in this symbiosis, we analysed the content of free amino acids in the same developmental groups studied in the transcriptome analysis. Our HPLC analysis revealed an accumulation of tyrosine in the late embryonic stages (Figure 4), coupled with a decrease in the aspartate content. It is worth noting that this amino acid is located upstream of the tyrosine pathway and is the amino group donor for phenylalanine synthesis (Figure 5). This observed biochemical phenotype corresponds to the induction, during pea aphid development, of the expression of specific genes coding for the enzymes of the final steps of the pathway. Our work demonstrates the existence of an insect specific gene transcription regulation of this key amino acid synthesis and, thus, complements our previous observations of a lack of significant gene expression regulation of the aromatic amino acid pathways in the symbiotic bacteria during embryonic development [53]. The accumulation of tyrosine during pea aphid development is probably facilitated by a total lack of the genes encoding for the tyrosine degradation pathway in this insect genome [23,29], a pathway that is present in 18 other arthropods (only one enzyme missing in *Apis mellifera*) with a sequenced and annotated genome (http://arthropodacyc.cycadsys.org/; Baa-Puyoulet *et al*. manuscript in preparation). Tyrosine has been previously identified as the most abundant free amino acid in body fluids in another aphid species, *Uroleucon ambrosiae* (Strecker) [64].

A recent RNAseq study on the pea aphid, by Hansen and Moran [31], demonstrated that several genes, coding for key enzymes complementary to the *B. aphidicola* metabolic repertoire, are highly expressed in the bacteriocytes (the aphid symbiotic cells) when compared to the rest of the body, excluding the embryonic compartment. In the present work, we identified two pea aphid genes (*ACYPI004243* and *ACYPI007803*) that are regulated during parthenogenetic and viviparous development to supply the growing embryos with tyrosine, a key precursor to the cuticle formation and sclerotization process. In fact, two other genes, *ACYPI008168* and *ACYPI009626*, both important for cuticle formation, are up-regulated in the late phases of the pea aphid embryonic development: they code, respectively, for the enzymes catalyzing the reactions that produce DOPA (EC 1.14.16.2) and dopamine (EC 4.1.1.28), starting from tyrosine (Figure 5). DOPA and dopamine participate in the melanization (darkening) and the sclerotization (hardening) of the insects’ cuticle formed by cuticular proteins and chitin [65,66]. The observed general increase in cuticular gene expression during the late phases of pea aphid embryo development (Additional file 10: Table S11) demonstrates that all components of this key developmental process in insects are active in the late stages of pea aphid parthenogenetic development.

Among all the transcriptionally regulated genes coding for enzymes that we were able to identify, the most interesting example is aspartate transaminase (E.C. 2.6.1.1). Among the four genes coding for this enzyme, only *ACYPI004243* showed significant increases in expression level during pea aphid development while the other three showed a constant expression, albeit at different levels for each gene (Figure 3). However, *ACYPI004243* showed a lower expression level in adult bacteriocytes in the RNAseq study, when compared with the entire body (excluding the embryonic compartment) [31]. Among the four genes coding for the enzyme aspartate transaminase (EC 2.6.1.1), *ACYPI000044* had the highest level of expression in the adult pea aphid bacteriocytes [31]. In our study, we detected a relatively higher expression of *ACYPI000044* in the embryos and larvae, in comparison to the other 3 genes, but the expression levels of this gene did not change during pea aphid development. The same absence of change in expression during development is true for the other two genes, *ACYPI006213* and *ACYPI003009*, which show different levels of expression (Figure 3C) that are consistent with the results of RNAseq experiments performed on adult bacteriocytes [31]. Although it is not possible to define the role of each gene coding for the enzymes with aspartate transaminase activity (EC 2.6.1.1) our data demonstrate, for the first time, that among these four genes only *ACYPI004243* expression levels increase during the parthenogenetic development of the pea aphid. This observation suggests that, in the pea aphid genome, *ACYPI004243* is controlled by a specific transcriptional regulation mechanism to respond to the need for phenylalanine and tyrosine synthesis during this important phase of development, with a particular link to cuticle formation.

## Conclusions

We have characterized, for the first time, the transcriptional profiles underlying distinct developmental groups in pea aphid parthenogenesis, thus providing new data for gene function annotation and novel hypothesis generation. Furthermore, as a result of the combination of transcriptional profile analysis with a biochemical approach, we were able to show a correlation between gene transcriptional changes at the enzyme level and the accumulation/decrease of corresponding amino acids, demonstrating an embryo-specific gene regulation in the phenylalanine and tyrosine pathways. The integrated metabolism of the pea aphid and its symbiotic bacteria, *B. aphidicola,* is far from being completely understood, but our study elucidates, in detail, the role of specific genes in tyrosine metabolism for cuticle formation during the parthenogenetic development of this symbiotic insect.

## Methods

### Aphid rearing and embryo isolation

A long-established parthenogenetic clone (LL01) of *A. pisum* was maintained at 21°C, with a 16 hour photoperiod, on *Vicia faba (*L. cv. Aquadulce*).* In order to have a supply of synchronised aphids and embryos, around one hundred mass-reared winged adults were maintained on young plants and removed after 24 h. The resulting apterous insects were maintained on *Vicia faba* plants for a nine-day period, until they reached the adult stage.

Embryos were dissected from synchronized parthenogenetic viviparous adult aphids, removing the ovariole sheath in two distinct ice-cold buffers depending on the subsequent analysis. For the total RNA extraction procedure, we used an RNase-free buffer composed of 35 mM Tris-HCl (pH 7.5), 25 mM KCl, 10 mM MgCl_2_, 250 mM sucrose, in 0.1% diethyl pyrocarbonate water. For the HPLC experiments, the buffer contained 162.75 mM KCl, 10 mM CaCl_2_, 25 mM MgCl_2_, 13.75 mM citric acid and 38.75 mM NaOH.

Following a stereoscopical analysis (Olympus IX-81, Olympus, France), embryos were classified according to their length and morphological characteristics into 3 groups: early embryos (EE) (≤ 0.4 mm), intermediate embryos (IE) (0.4 to 0.8 mm) and late embryos (LE) (> 0.8 mm) corresponding, respectively, to the developmental stages ≤ 15, 16-18 and 19-20 as described by Miura *et al.*[13] (Table 1). These groups were chosen because it was easy to distinguish and separate them, during the dissection step, on the basis of well recognizable external morphological criteria. These same developmental stages were also chosen in a previous work on *B. aphidicola* transcriptome analysis during pea aphid development [53], thus allowing us to make a direct comparison of the transcriptomic data from both the insect host and the symbiotic bacteria.

To obtain synchronized early and late L1, viviparous adults were maintained on young plants for 6 hours. The early L1 (aged from 0 to 6 h) were collected and, for the late L1 (aged from 13 to 19 h), after having discarded the adults, larvae were maintained for a further 13 h on the plants prior to collection. For L1 aged from 0 to 24 h, viviparous adults were maintained on young plants for 24 hours and the resulting L1 were collected (Table 1).

### RNA extraction

Total RNA was prepared using the RNeasy mini kit (Qiagen, Hilden, Germany). Three independent extractions were prepared for each group starting from 60 embryos for the EE group, 30 embryos for both the IE and LE groups, and 30 larvae for the L1 group (0-24 h). It is worth noting that the RNA extractions for the microarray and for the qRT-PCR experiments were performed independently, thus the qRT-PCR data constitute a full biological experimental replicate of the microarray results. For the qRT-PCR, the extraction also included a step of DNase treatment (RNase-Free DNase Set, Qiagen). Total RNA concentration and quality were initially checked using the NanoDrop® ND-1000 spectrophotometer (NanoDrop Technologies, Wilmington, DE, USA) and samples had to meet the following quality parameters: A260/A280 ≥ 1.8 and A260/A230 ≥ 1.8, in order to be used in the subsequent analysis. The RNA samples were then run using the Agilent RNA 6000 Nano Kit on the Agilent 2100 Bioanalyzer (Agilent Technologies, Palo Alto, CA) to check their integrity. Degraded samples appeared as significantly lower intensity traces, with the main peak area shifted to the lower molecular weights, and they typically exhibited much more noise on the trace. Only good quality samples were used for the subsequent analysis.

### Amplification of mRNA and cDNA synthesis

A limited amount of total RNA was available and we used a T7 RNA polymerase based linear amplification method that has been shown to have no systematic influence on microarray transcription profiles [67]. We used the MessageAmp™ II aRNA Amplification kit (Ambion, Austin, TX, USA) that preferentially amplifies eukaryotic mRNA. Following the manufacturer’s instructions, RNA amplification included five steps: (1) a reverse-transcription using 1 μg of total RNA and 1 μl of T7-oligo(dT) primers (100 ng/μl), (2) a second strand cDNA synthesis, (3) a cDNA purification on a DNA filter cartridge, (4) an *in vitro* transcription and (5) an amplified RNA purification on an aRNA Filter Cartridge. Double strand cDNA was prepared using the Superscript II kit (Invitrogen, Paisley, UK), as recommended by NimbleGen in the NimbleChip™ Arrays User’s Guide for gene expression analysis. Starting with 10 μg of aRNA, the samples were processed according to the manufacturer’s instructions, including these four steps: (1) an initial cDNA synthesis using random primers, (2) a second strand synthesis, (3) an RNase A clean-up, and (4) a cDNA precipitation. For each sample, the integrity of the aRNA and cDNA was checked using the Agilent RNA 6000 Nano Kit on the Agilent 2100 Bioanalyzer. Only good quality samples were retained for the microarray experiments.

### Microarray experiments and data collection

The “INRA-BF2I_A.pisum_Nimblegen-ACYPI_4x72k_v1” microarray for the pea aphid was developed in collaboration with NimbleGen using the pea aphid genome v1.0 assembly [23]. This NimbleGen 385 K 4-plex (4 × 72,000 probes) high-density array can accommodate four samples that are hybridized onto a section of the array containing 72,000 60-mers oligonucleotide probes, representing 24,011 pea aphid transcripts (corresponding to 23,855 genes). The microarray design is deposited in the ArrayExpress database (http://www.ebi.ac.uk/arrayexpress/) and the array is available to the International Aphid Genomics Consortium community. On the arrays, 185 probe sets recognise more than one gene: these ACYPI probe sets are labelled with an asterisk (*) in all of the Results tables and they are listed in full in Table S1E. Labelling (using the NimbleGen One-Color DNA Labelling Kits and Cy3 Random Nonamers), hybridization on the arrays (at 42°C for 16-20 hours) and scanning (using MS 200 Microarray Scanner and the MS 200 Data Collection Software) were carried out by Roche NimbleGen, as described in the NimbleGen arrays user’s guide for gene expression arrays, and they provided the final data files. All the transcriptomic data obtained are available in the ArrayExpress database (http://www.ebi.ac.uk/arrayexpress/).

### Microarray data analysis

Microarray data were normalized, using the RMA method [68], and then transformed into log2. A one-way between groups ANOVA analysis was performed using the Limma package in the R software [69]: two by two comparisons were performed to identify any differentially expressed genes between the different stages of development (EE-IE; IE-LE; LE-L1). The non-parametric p-values were estimated using 1000 sample permutations and further adjusted using the Benjamini and Hochberg method [70], to limit the number of false positives by a control of the False Discovery Rate (FDR). For this study we chose an FDR lower than 0.05. A gene was considered significant if its adjusted p-value was lower than 0.05 and if it showed a two-fold change in the considered contrast (see Additional file 1: Table S1 for a detailed list of significantly expressed genes). All HCL (Hierarchical Clustering) analyses were carried out using the TMeV software [71], applying the average linkage method with euclidean distance and no leaf order optimization. Gene Ontology analysis was carried out using the Blast2GO software [72] to perform the GOSSIP test ([73]; http://gossip.gene-groups.net).

### Validation of microarray data by qRT-PCR

Total RNA was reverse-transcribed in cDNA using the SuperScript™ III First-Strand Synthesis System for RT-PCR (Invitrogen, Paisley, UK), with random primers, according to the manufacturer’s instructions. This protocol involved three principal steps: (1) an incubation for 5 min at 65°C, (2) a reverse transcription using 1 μg of total RNA and including three incubations (25°C, 50°C and 85°C), (3) an RNA matrix degradation using RNase H. Primers to target transcripts (Additional file 2: Table S12) were designed with the Oligo7 software [74], except those used for the genes *ACYPI009127* and *ACYPI001858* which were taken from Brisson *et al.*[75]. Real-time PCR was performed in 96-well plates with a LightCycler 480 instrument (Roche diagnostics, Meylan, France). Either 2.5 μl of cDNA (at around 1 μg/μl), diluted at 1/5, or water (for negative control reactions) were used in a total PCR reaction final volume of 10 μl (reagents used from the LightCycler FastStart DNA Master SYBR green I kit by Roche). Amplification conditions were as follows: 95°C for 5 min and then 45 cycles of 95°C for 15 s, 53°C for 15 s, and 72°C for 1 min 10 s. An internal standard curve was generated for each gene using serial dilutions (from 2000 to 0.0002 μg/μl) of purified PCR products amplified from a pool of cDNA. The PCR reaction, to prepare the control sample for the standard curve, was carried out starting from 1 μl of reverse transcription product using UptiTherm DNA Polymerase (Interchim, Montluçon, France), according to the following protocol: activation of Taq DNA polymerase at 95°C for 5 min, followed by 34 three-step amplification cycles consisting of 30 s denaturation at 95°C, 45 s annealing at 53°C, and 45 s of extension at 72°C. For the data normalization, two genes were tested in the different developmental stage groups analysed here: *actin* (*ACYPI000064*) and *rpl32* (*ACYPI000074*). Real-time RT-PCR data were analysed using the *BestKeeper*^*©*^ software tool [76] and the *actin* gene was retained as the best candidate for data normalization. An analysis of the quantitative RT-PCR data was performed using the REST software ([54]; http://rest.gene-quantification.info/). The relative expression ratio of each target gene was calculated by comparing the tested condition against the control condition, and also relative to the normalization gene. More precisely, this ratio (R) was calculated taking into account the real-time PCR efficiency of each gene (E) and the crossing point difference (ΔCP) of a test condition (IE, LE or L1), as compared to the reference condition (EE, IE or LE according to the comparison (EE-IE, IE-LE, LE-L1)), and expressed in comparison to the normalization gene (*actin*) using the following model [77].

$$R=\frac{{\left(E_{\mathrm{target}}\right)}^{\Delta {\mathrm{CP}}_{\mathrm{target}}\left(\mathrm{control}-\mathrm{sample}\right)}}{{\left(E_{\mathrm{reference}}\right)}^{\Delta {\mathrm{CP}}_{\mathrm{reference}}\left(\mathrm{control}-\mathrm{sample}\right)}}$$

### Sample preparation for free amino acid analysis

For the quantification of free amino acids, we used 20 embryos (Early or Intermediate groups), 15 embryos (Late group) or 15 L1 (0-6 h or 13-19 h) per replicate and the analyses were performed on at least four independent replicates. Samples were crushed in 320 μl of ultra-pure water with a known quantity of norvaline used as the internal standard. 200 μl of this crude homogenate were used for free amino acid analyses. Free amino acids were extracted from crude homogenate with trichloroacetic acid (TCA, 5% w/v final concentration), maintained at room temperature for 2 h, vortexed every 30 min, and then centrifuged (10,000 *g* for 10 min at 4°C). TCA was eliminated from the supernatant by chloroform/water partition (three successive extractions with 400 μl of chloroform), and the final aqueous supernatant was dried under vacuum. All samples were stored at -20°C, and then mixed with 80 μl of HCl 0.1 N for amino acid analysis.

### Amino acid analysis and quantification

Amino acid analysis was performed by HPLC (Agilent 1100; Agilent Technologies, Massy, France) with a guard cartridge and a reverse phase C18 column (Zorbax Eclipse-AAA 3.5 μm, 150 × 4.6 mm, Agilent Technologies), according to the procedure specifically developed for this system [78]. Prior to injection, the sample was buffered with borate at pH 10.2, and primary or secondary amino acids were derivatized with ortho-phthalaldehyde (OPA) or 9-fluorenylmethyl chloroformate (FMOC), respectively. The derivatization process, at room temperature, was automated using the Agilent 1313A autosampler. Separation was carried out at 40°C, with a flow rate of 2 ml/min, using 40 mM NaH_2_PO_4_ (eluent A, pH 7.8, adjusted with NaOH) as the polar phase and an acetonitrile/methanol/water mixture (45/45/10, v/v/v) as the non-polar phase (eluent B). A gradient was applied during chromatography, starting with 20% of B and increasing to 80% at the end. Detection was performed by a fluorescence detector set at 340 and 450 nm of excitation and emission wavelengths, respectively (266/305 nm for proline). These conditions do not allow for the detection and quantification of cystine and tryptophan, so only 18 amino acids were quantified. For this quantification, norvaline was used as the internal standard and the response factor of each amino acid was determined using a 250 pmol/μl standard mix of amino acids. The software used was the ChemStation for LC 3D Systems (Agilent Technologies).

The comparison of the relative concentrations of free amino acids in the aphid at the various developmental stages (expressed as % of total free amino acids) was performed, after angular transformation to normalize data, using a one-way ANOVA followed by a two by two comparison (Student-Newman-Keuls test).

### Sequence analyses

The four protein sequences (ACYPI006213-PA, ACYPI003009-PA, ACYPI004243-PA and ACYPI000044-PA) for the aspartate transaminase enzymatic activity (E.C. 2.6.1.1) were obtained from PhylomeDB [59], together with their corresponding arthropod orthologs and paralogs. The data set was completed with other homologous proteins, extracted from the UniProt database [60], using a stringent e-value (= 10^-6^) for the BlastP search. Amino acid sequences were then aligned using the software MUSCLE [79] embedded in the phylogeny software SeaView 4.0 [80]. A phylogenetic tree was drawn in order to remove non-homologous proteins and redundant sequences and to reduce the species number in some taxonomic groups of the tree. The alignment was then recalculated and manually corrected. Trees were calculated using Poisson and Kimura distances, with the BIONJ heuristic [81], and by applying maximum likelihood estimations. The tree was rooted, by default, using the longest branch. The mitochondrial target analysis was performed using MITOPROT, an analysis tool for the prediction of mitochondrial targeting sequences ([82]; http://ihg.gsf.de/ihg/mitoprot.html).

## Abbreviations

EC: Enzyme commission (a numerical classification scheme for enzymes, based on the chemical reactions they catalyse); PCR: Polymerase chain reaction; HPLC: High-performance liquid chromatography.

## Competing interests

The authors declare that they have no competing interests.

## Authors’ contributions

The authors have made the following declarations concerning their contributions. AR, GF, HC, FC and SC conceived and designed the experiments. AR, KG, GD, NB and MR performed the experiments. AR, GF, PBP, HC, FC and SC analysed the data. PBP, PS and YR contributed reagents/materials/analysis tools. AR, GF, PBP, HC, FC and SC contributed to text, tables and figures in the paper. AR, FC and SC wrote the paper. All authors read and approved the final manuscript.

## Supplementary Material

Additional file 1: Table S1 List of genes differentially expressed in the three comparisons. A summary of all significant genes identified in this study, with their fold change differences, using a one-way between groups ANOVA for the three pair-wise comparisons: **A)** EE *vs.* IE, **B)** IE *vs.* LE, and **C)** LE *vs.* L1. **D)** Venn diagrams of gene lists of the two contrasts: panel (I) = list A *vs.* list B, panel (II) = list B *vs.* list C. **E)** List of genes with shared probes with other genes. Click here for file

Additional file 2: Table S2, S8, S9, S10, S12. Table S2 Microarray data validation by qRT-PCR. **Table S8.** Relative free amino acid contents during pea aphid development. **Table S9.** PRIAM analysis results summary. **Table S10.** MITOPROT analysis results summary. **Table S12.** Oligonucleotide primers used for qRT-PCR. Click here for file

Additional file 3: Table S3 List of developmental genes differentially expressed in the three comparisons. A summary of all developmental genes showing significant differential expression for the three pair-wise comparisons **A)** EE *vs.* IE, **B)** IE *vs.* LE and **C)** LE *vs.* L1. **D)** Developmental genes showing significant expression changes and belonging to specific functional classes (homeobox-containing genes and major components of signalling pathways): in red, increasing expression during development and, in blue, decreasing expression during development. Click here for file

Additional file 4: Table S4 List of functional classes enriched in significant variant genes for the three comparisons. Result of GOSSIP ([73]; http://gossip.gene-groups.net) analysis performed using Blast2GO ([72]; http://www.blast2go.com/) on the gene showing significant differential expression for the three pair-wise comparisons **A)** EE *vs.* IE, **B)** IE *vs.* LE and **C)** LE *vs.* L1. Click here for file

Additional file 5: Table S5 List of significant variant genes involved in metabolism from AcypiCyc. Result of the metabolism analysis performed in the AcypiCyc database ([56]; http://acypicyc.cycadsys.org/) on the list of significant variant genes for the three pair-wise comparisons **A)** EE *vs.* IE, **B)** IE *vs.* LE and **C)** LE *vs.* L1. Click here for file

Additional file 6: Table S6 List of significant variant genes involved in the biosynthesis or the degradation of amino acids. Summary table of annotated amino acids metabolism genes (modified from [29]) showing differential gene expression in the three pair-wise comparisons EE *vs.* IE, IE *vs.* LE and LE *vs.* L1. Click here for file

Additional file 7: Table S7 List of amino acid transporter genes and their gene expression data. **A)** Expressions of amino acid transporter genes (as annotated by Price *et al.*[57]) with, in bold, the genes showing a significant gene expression change during development. Data are presented as log2 intensities for each replicate. **B)** Summary table of transporter genes showing differential gene expression in the three pair-wise comparisons EE *vs.* IE, IE *vs.* LE and LE *vs.* L1. Click here for file

Additional file 8: Figure S1 EC 2.6.1.1 enzymatic activity proteins alignment. Alignment of the 4 proteins having the aspartate transaminase enzymatic activity (EC 2.6.1.1) in the pea aphid, as performed using ClustalW method in MUSCLE [79], available online at the European Bioinformatics Institute (http://www.ebi.ac.uk/Tools/msa/muscle/). Click here for file

Additional file 9: Figure S2 Phylogenetic analysis of genes coding for the proteins with EC 2.6.1.1 enzymatic activity. Red circles represent duplication events and brown rectangles represent the corresponding paralogous families, named using consensual annotation. Accession numbers are those from the PhylomeDB (http://phylomedb.org/) or UniProtKB/Swiss-Prot (http://www.uniprot.org/), followed by species names (*A. pisum* paralogs are written in red). Bootstrap values over 50% are given at each node of the tree (1000 replicates). The tree (Poisson distance, BIONJ heuristic) was rooted by its longest branch (i.e., the bacterial branch). Scale 0.1 amino acid substitutions by position (257 informative sites). Although the three clades, named AATC2, AATC1 and AATC, are well supported by bootstrap values and always conserved, their respective positioning is not clear, varying according to the tree-building methods used, as illustrated by the very short branches separating them. The number of coding exons for each gene (when available) is given in square brackets after the AATM protein names. Click here for file

Additional file 10: Table S11 Cuticle precursor and cuticular protein genes. Expression levels of the mRNA (log2 intensities for each replicate) and fold-change differences (in log2) for mRNA showing differential expression in the three pair-wise comparisons EE *vs.* IE, IE *vs.* LE and LE *vs.* L1: in red, increasing expression during development and, in blue, decreasing expression during development. **A)** Genes coding for the enzymes involved in the synthesis of DOPA and dopamine starting from tyrosine. **B)** Genes coding for cuticular proteins. Click here for file
