# Supplementary material for: Tyrosine pathway regulation is host-mediated in the pea aphid symbiosis during late embryonic and early larval development
Source: BMC Genomics. 2013 Apr 10;14:235. doi: 10.1186/1471-2164-14-235 (PMC3660198; doi:10.1186/1471-2164-14-235)
Supplement: Additional file 2: Table S2, S8, S9, S10, S12. Table S2 — Microarray data validation by qRT-PCR. Table S8. Relative free amino acid contents during pea aphid development. Table S9. PRIAM analysis results summary. Table S10. MITOPROT analysis results summary. Table S12. Oligonucleotide primers used for qRT-PCR. [file 1471-2164-14-235-S2.docx]

**Additional file 2**

**Table S2. Microarray data validation by qRT-PCR.**

Eight target genes, belonging to four different functional classes, were tested by qRT-PCR. The differential gene expression variation (means of three independent experiments) for each of these target genes was calculated by comparing the four developmental groups, two by two and relative to the normalization gene actin (*ACYPI000064*), using the REST software [53]. The thresholds for significant differentially expressed genes were fixed, respectively, at +1 and - 0.5 (expressed as log2 and both corresponding to a two fold change variation) for up- and down-regulation. The data on microarray experiments are considered significantly differentially expressed using the following criteria: an adjusted p-value lower than 0.05 and a two-fold change in the considered contrast (see Methods for details on the analysis).

| **Gene** | | **Annotation** | **qRT-PCR data** | | |  | **microarray data** | | |
| --- | --- | --- | --- | --- | --- | --- | --- | --- | --- |
|  | |  | Ratio group | Log2 expression ratio | Regulation |  | Comparison during development | Log2 fold change difference | Regulation |
| ***Developmental genes*** | | |  |  |  |  |  |  |  |
| ACYPI009127 | | *dpp3* | IE/EE | **-1.80** | **down** |  | EE-IE | **-1.64** | **down** |
|  |  |  | LE/IE | 0.62 | invariant |  | IE-LE | -0.66 | invariant |
|  |  |  | L1/LE | -0.41 | invariant |  | LE-L1 | -0.26 | invariant |
| ACYPI001858 | | *ubx* | IE/EE | **-1.02** | **down** |  | EE-IE | -0.59 | invariant |
|  |  |  | LE/IE | **1.42** | **up** |  | IE-LE | -0.36 | invariant |
|  |  |  | L1/LE | **-0.54** | **down** |  | LE-L1 | **-1.12** | **down** |
| ACYPI009481 | | *wnt7* | IE/EE | -0.09 | invariant |  | EE-IE | -0.04 | invariant |
|  |  |  | LE/IE | **-0.56** | **down** |  | IE-LE | **-2.04** | **down** |
|  |  |  | L1/LE | **1.21** | **up** |  | LE-L1 | **1.47** | **up** |
| ***Amino acid pathways*** | | |  |  |  |  |  |  |  |
| ACYPI004243 | aspartate aminotransferase | | IE/EE | 0.63 | invariant |  | EE-IE | 0.06 | invariant |
|  |  |  | LE/IE | **2.60** | **up** |  | IE-LE | **2.37** | **up** |
|  |  |  | L1/LE | **3.35** | **up** |  | LE-L1 | **2.91** | **up** |
| ACYPI007803 | phenylalanine-4-monooxygenase | | IE/EE | **3.60** | **up** |  | EE-IE | 0.36 | invariant |
|  |  |  | LE/IE | **1.22** | **up** |  | IE-LE | **2.36** | **up** |
|  |  |  | L1/LE | -0.01 | invariant |  | LE-L1 | -1.05 | invariant |
| ACYPI008168 | tyrosine-3-monooxygenase | | IE/EE | 0.68 | invariant |  | EE-IE | 0.08 | invariant |
|  |  |  | LE/IE | **8.06** | **up** |  | IE-LE | **5.34** | **up** |
|  |  |  | L1/LE | **-3.80** | **down** |  | LE-L1 | **-1.59** | **down** |
| ***Cuticular genes*** | | |  |  |  |  |  |  |  |
| ACYPI009491 | | cuticular protein 111 (RR3 family) | IE/EE | 0.99 | invariant |  | EE-IE | 0.65 | invariant |
|  |  |  | LE/IE | **5.27** | **up** |  | IE-LE | **2.38** | **up** |
|  |  |  | L1/LE | **1.22** | **up** |  | LE-L1 | **1.65** | **up** |
| ***Transporters*** | | |  |  |  |  |  |  |  |
| ACYPI004721 | | oligopeptide transporter | IE/EE | **9.61** | **up** |  | EE-IE | **4.10** | **up** |
|  |  |  | LE/IE | **2.44** | **up** |  | IE-LE | **2.82** | **up** |
|  |  |  | L1/LE | **-1.06** | **down** |  | LE-L1 | -0.88 | invariant |

**Table S8. Relative free amino acid contents during pea aphid development.**

Relative free amino acid contents of the EE, IE and LE embryos and the early and late first larval stages (L1). The proportion of each amino acid is expressed in % ± SE of total free amino acids (n = 4). Comparisons between the various developmental stages were performed, after angular transformation, by a one-way ANOVA (significance expressed by the p-value) followed by a two by two comparison (Student-Newman-Keuls test). Within a row, percentages with the same letter are not significantly different (*P* < 0.05).

|  | **EE** | **IE** | **LE** | **L1 early** | **L1 late** | **p-value** |
| --- | --- | --- | --- | --- | --- | --- |
| Asp | 16.4 ± 0.7 ^a^ | 8.1 ± 0.3 ^b^ | 2.1 ± 0.3 ^c^ | 0.7 ± 0.1 ^d^ | 0.8 ± 0.3 ^d^ | < 0.0001 |
| Glu | 29.6 ± 0.6 ^b^ | 35.2 ± 0.4 ^a^ | 23.4 ± 0.5 ^c^ | 16.9 ± 0.7 ^d^ | 17.8 ± 1.2 ^d^ | < 0.0001 |
| Asn | 12.1 ± 0.6 ^a^ | 7.9 ± 0.8 ^a^ | 8.7 ± 1.1 ^a^ | 6.7 ± 0.2 ^a^ | 8.9 ± 2.6 ^a^ | 0.09 |
| Ser | 4.9 ± 0.3 ^a^ | 4.3 ± 0.1 ^a^ | 3.7 ± 0.2 ^ab^ | 2.7 ± 0.2 ^b^ | 3.9 ± 0.8 ^ab^ | 0.003 |
| Gln | 3.8 ± 0.3 ^b^ | 3.4 ± 0.4 ^b^ | 5.4 ± 1.1 ^b^ | 6.7 ± 0.7 ^ab^ | 12.3 ± 3.5 ^a^ | 0.004 |
| His | nd | nd | 2.3 ± 0.1 | 3.3 ± 0.4 | 1.9 ± 0.2 |  |
| Gly | 3.8 ± 0.4 ^ab^ | 4.4 ± 0.2 ^ab^ | 5.2 ± 0.3 ^a^ | 3.5 ± 0.2 ^b^ | 4.7 ± 0.7 ^ab^ | 0.02 |
| Thr | 2.5 ± 0.2 ^a^ | 2.3 ± 0.2 ^a^ | 1.7 ± 0.1 ^b^ | 1.1 ± 0.1 ^c^ | 2.1 ± 0.1 ^ab^ | < 0.0001 |
| Arg | 2.3 ± 0.5 ^b^ | 2.7 ± 0.2 ^b^ | 4.4 ± 0.4 ^a^ | 5.2 ± 0.2 ^a^ | 5.2 ± 0.5 ^a^ | < 0.0001 |
| Ala | 11.1 ± 0.8 ^b^ | 15.5 ± 0.3 ^a^ | 14.1 ± 0.8 ^ab^ | 15.6 ± 0.7 ^a^ | 14.1 ± 1.1 ^ab^ | 0.004 |
| Tyr | 1.9 ± 0.3 ^d^ | 2.1 ± 0.1 ^d^ | 12.1 ± 1.0 ^b^ | 17.6 ± 1.4 ^a^ | 6.8 ± 1.1 ^c^ | < 0.0001 |
| Val | 3.5 ± 0.2 ^ab^ | 3.7 ± 0.1 ^a^ | 3.2 ± 0.3 ^ab^ | 3.4 ± 0.2 ^ab^ | 2.7 ± 0.2 ^b^ | 0.04 |
| Met | nd | nd | 0.80 ± 0.04 | 0.57 ± 0.03 | 0.61 ± 0.07 |  |
| Phe | 1.5 ± 0.2 ^b^ | 1.9 ± 0.3 ^ab^ | 2.9 ± 0.3 ^a^ | 2.8 ± 0.3 ^a^ | 1.6 ± 0.2 ^b^ | 0.002 |
| Ile | 1.6 ± 0.04 ^b^ | 2.2 ± 0.2 ^a^ | 1.8 ± 0.1 ^ab^ | 1.4 ± 0.1 ^b^ | 1.5 ± 0.1 ^b^ | 0.001 |
| Leu | 2.0 ± 0.04 ^a^ | 2.0 ± 0.1 ^a^ | 2.2 ± 0.1 ^a^ | 1.2 ± 0.1 ^a^ | 3.6 ± 2.6 ^a^ | 0.6 |
| Lys | 0.4 ± 0.4 ^c^ | 0.8 ± 0.2 ^bc^ | 2.6 ± 0.3 ^ab^ | 4.8 ± 1.0 ^a^ | 4.2 ± 0.8 ^a^ | < 0.0001 |
| Pro | 2.6 ± 0.4 ^b^ | 3.4 ± 0.5 ^b^ | 3.5 ± 0.1 ^b^ | 5.9 ± 0.6 ^a^ | 7.2 ± 0.7 ^a^ | < 0.0001 |

**Table S9. PRIAM analysis results summary.**

Summary of the results of the analysis of the four proteins ACYPI000044-PA, ACYPI003009-PA, ACYPI004243-PA and ACYPI006213-PA performed using PRIAM [57] (http://priam.prabi.fr/); only the BLAST hits against PRIAM profiles with an E-value < 0.05 are shown.

| **Protein ID** | **PRIAM profile** | **PRIAM profile**  **E.C. number** | **E-value** |
| --- | --- | --- | --- |
| ACYPI000044-PA | PRI001238 | 2.6.1.1 | 1e-57 |
|  | PRI002923 | 2.6.1.5 | 1e-13 |
|  | PRI001269 | 2.6.1.57 | 1e-12 |
| ACYPI003009-PA | PRI001238 | 2.6.1.1 | 2e-46 |
|  | PRI001269 | 2.6.1.57 | 2e-09 |
|  | PRI002923 | 2.6.1.5 | 1e-07 |
|  | PRI001277 | 2.6.1.9 | 0.027 |
| ACYPI004243-PA | PRI001238 | 2.6.1.1 | 9e-48 |
|  | PRI001269 | 2.6.1.57 | 2e-14 |
|  | PRI002923 | 2.6.1.5 | 5e-10 |
|  | PRI003678 | 2.6.1.83 | 0.007 |
| ACYPI006213-PA | PRI001238 | 2.6.1.1 | 4e-59 |
|  | PRI002923 | 2.6.1.5 | 2e-13 |
|  | PRI001269 | 2.6.1.57 | 2e-10 |
|  | PRI003678 | 2.6.1.83 | 3e-04 |
|  | PRI001277 | 2.6.1.9 | 0.007 |

**Table S10. MITOPROT analysis results summary.**

Summary of the results of the analysis of the four proteins ACYPI000044-PA, ACYPI003009-PA, ACYPI004243-PA and ACYPI006213-PA performed using MITOPROT ([81]; (http://ihg.gsf.de/ihg/mitoprot.html).

| **Protein ID** | **Cleavage site** | **Cleaved sequence** | **PROBABILITY of**  **export to mitochondria** |
| --- | --- | --- | --- |
| ACYPI000044-PA | 23 | MSMSKCHQLSLFVCKQQKNLRM | 0.4143 |
| ACYPI003009-PA | - | not predictable | 0.0045 |
| ACYPI004243-PA | - | not predictable | 0.0107 |
| ACYPI006213-PA | - | not predictable | 0.0254 |

**Table S12. Oligonucleotide primers used for qRT-PCR.**

The Forward (F) and the Reverse (R) primers, and the DNA fragment length obtained after RT-PCR, are all listed for each primer pair.

| **ACYPI mRNA ID** | **Primer** | **Sequences** | **Amplicon length (bp)** |
| --- | --- | --- | --- |
| ACYPI009127-RA | Forward (F) | 5’-CGA-TCA-AGT-ACT-ACG-GAA-TGG-AG-3’ | 95 |
|  | Reverse (R) | 5’-ATT-CTG-AGA-ACA-GGT-TTC-GAT-AGG-3’ |  |
| ACYPI001858-RA | Forward (F) | 5’-GCC-ATT-GCA-GGT-CAG-TCT-CT-3’ | 119 |
|  | Reverse (R) | 5’-GCG-AGT-CAG-GTA-GTG-GTT-GG-3’ |  |
| ACYPI007803-RA | Forward (F) | 5’-TTC-ACG-ACA-AAA-AAC-ATT-CTT-TC-3’ | 124 |
|  | Reverse (R) | 5’-AAA-TTA-AAC-GCA-GTA-ACG-AAC-3’ |  |
| ACYPI004721-RA | Forward (F) | 5’-CTG-TAT-AAG-ATC-ATT-AAA-CCC-3’ | 188 |
|  | Reverse (R) | 5’-AAT-AAA-ACA-TCC-AAC-GC-3’ |  |
| ACYPI004243-RA | Forward (F) | 5’-AAT-AGG-TAT-TAA-AAT-AGA-CTG-3’ | 193 |
|  | Reverse (R) | 5’-AAT-AGG-TAT-TAA-AAT-AGA-CTG-3’ |  |
| ACYPI009481-RA | Forward (F) | 5’-GTC-GGC-GGT-GGT-AAT-C-3’ | 168 |
|  | Reverse (R) | 5’-CCT-GTC-GCA-GTA-GTT-AGG-TGA-3’ |  |
| ACYPI009491-RA | Forward (F) | 5’-CCA-AGA-TGA-GTT-CGG-TCA-GTA-3’ | 133 |
|  | Reverse (R) | 5’-GTA-GTG-TTG-GGT-CTG-TCC-GT-3’ |  |
| ACYPI008168-RA | Forward(F) | 5’-AAG-GGC-TAC-TCT-GGT-ATT-GC-3’ | 238 |
|  | Reverse(R) | 5’-GTC-TTC-GGC-TAG-AAT-CGT-AAT-3’ |  |
| ACYPI000064-RA | Forward (F) | 5’-AAG-TTA-TCA-CAA-TCG-GAA-ATG-3’ | 196 |
|  | Reverse (R) | 5’-GGC-AAT-ACC-AGG-GTA-CAT-3’ |  |
