# Supplementary figures and images for: Tyrosine pathway regulation is host-mediated in the pea aphid symbiosis during late embryonic and early larval development
Source: BMC Genomics. 2013 Apr 10;14:235. doi: 10.1186/1471-2164-14-235 (PMC3660198; doi:10.1186/1471-2164-14-235)

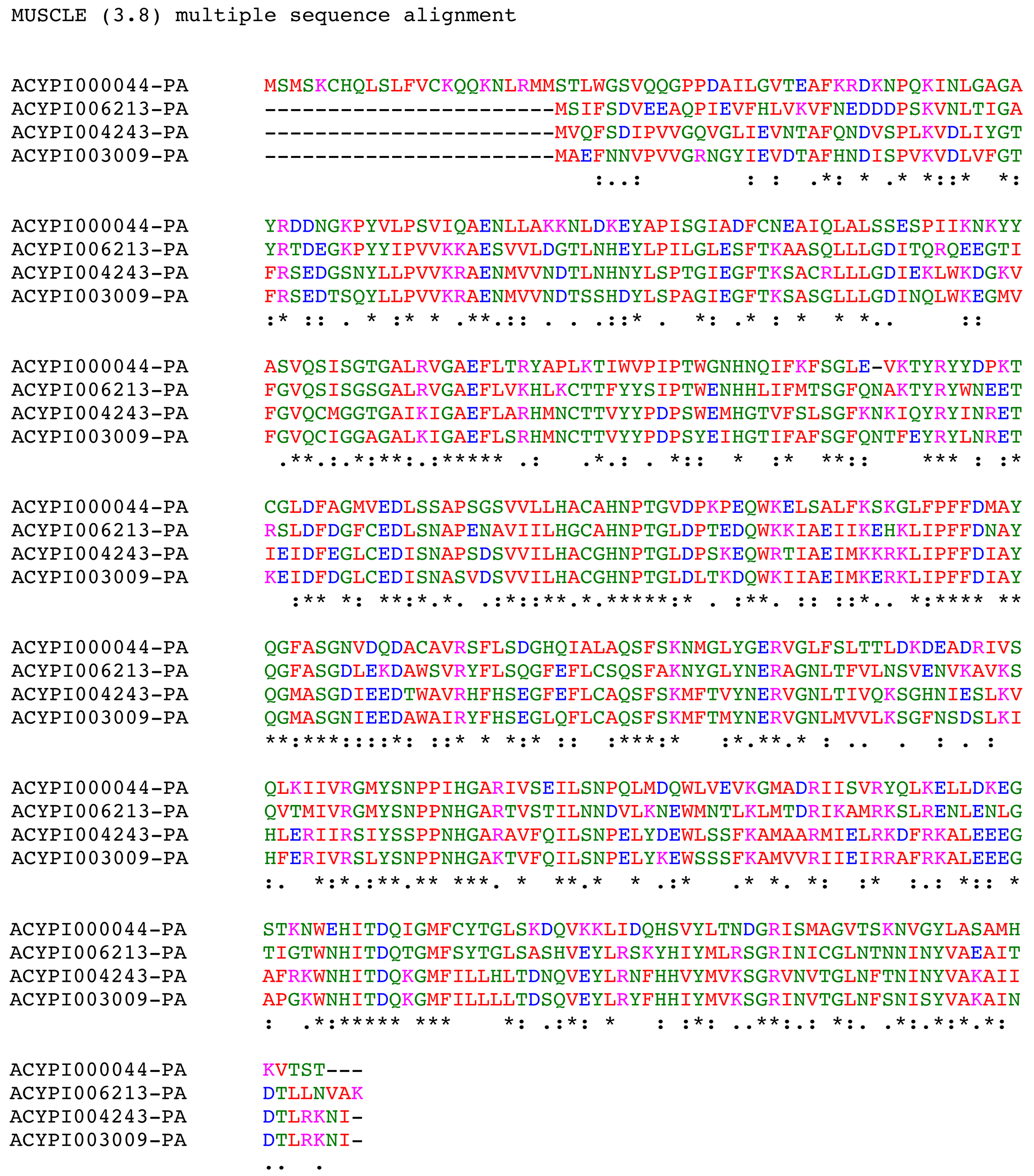

Supplement: Additional file 8: Figure S1 — EC 2.6.1.1 enzymatic activity proteins alignment. Alignment of the 4 proteins having the aspartate transaminase enzymatic activity (EC 2.6.1.1) in the pea aphid, as performed using ClustalW method in MUSCLE [79], available online at the European Bioinformatics Institute (http://www.ebi.ac.uk/Tools/msa/muscle/). [file 1471-2164-14-235-S8.tiff]
